# Implications of the COVID-19 Pandemic on Adult Day Services

**DOI:** 10.1093/geroni/igab046.420

**Published:** 2021-12-17

**Authors:** Katherine Marx, Laura Gitlin, Tina Sadarangani

## Abstract

Adult day service (ADS) centers serve an important role in care provision for people living with dementia (PLWD). These programs provide socialization, activities, and access to many therapies for PLWD. Additionally, they offer respite to family caregivers so they can work, run errands, and recharge. In March 2020, like much of the nation, ADS sites across the nation were shut down. This left many PLWD and their caregivers without access to the services they depended on to manage day to day care. It also left many sites without a revenue source to pay employees and maintain buildings. Almost a year later, many states have still not reopened ADS and sites that have reopened have done so with a lower census, increased costs, and the lingering fear of a second closure. Much focus has been on the care of older adults in nursing homes or other residential long-term care settings but the challenges of ADS and the people they serve has been mostly ignored. The purpose of this symposium is to highlight the implications of the COVID-19 pandemic on ADS centers. Holly Dabelko-Schoeny will present data gathered from ADS Centers across Ohio. Lauren Parker, will then present data from ADS sites across the United States that examines the effect of COVID-19 on closures and programming during the closures. Katherine Marx will present the effect of ADS closures on family caregivers of persons living with dementia. Finally, Joseph Gaugler will explore this from a policy perspective and provide recommendations moving forward.

